# Denture Misadventure: The Tooth About Your Colon

**DOI:** 10.7759/cureus.3890

**Published:** 2019-01-15

**Authors:** Rajesh Essrani, Patrick Hickey, Hiral Shah

**Affiliations:** 1 Internal Medicine, Lehigh Valley Health Network, Allentown, USA; 2 Gastroenterology, Lehigh Valley Health Network, Allentown, USA

**Keywords:** foreign body, endoscopy, colonoscopy

## Abstract

Foreign body ingestion occurs mainly in a mentally impaired person, but rarely in healthy adults. A 48-year-old male was admitted with a recurrence of previously diagnosed right knee septic arthritis and accidentally ingested his dental bridge which was removed from a proximal ascending colon with a colonoscopy.

## Introduction

Foreign body ingestion occurs mainly in a mentally impaired person, but rarely in healthy adults. Most ingested foreign bodies (80%-90%) pass without the need for intervention [1]. Endoscopic intervention is required in 10% to 20% of patients, and surgical intervention is required in less than 1% of the patients [2-3]. We present a case of accidental dental bridge ingestion in a 48-year-old male which was removed by colonoscopy.

## Case presentation

**Figure 1:**
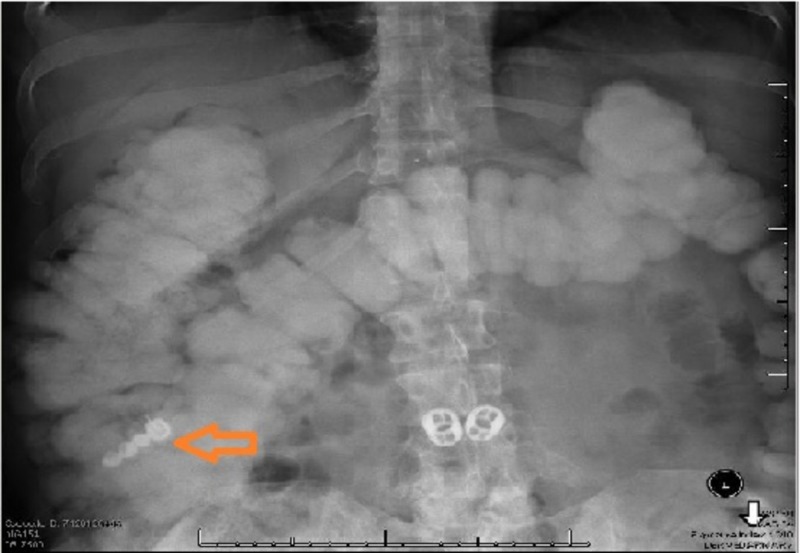
Abdominal X-ray showing the foreign body overlying the right abdomen; it could be within the proximal transverse colon, or could be within the right colon

A 48-year-old man was admitted with a recurrence of previously diagnosed right knee septic arthritis requiring multiple surgical interventions and treatment with high-dose narcotic analgesia. During his hospitalization, he attempted to place his upper left dental bridge, but he accidentally ingested it. He noted that the partial dental appliance had an exposed screw. On evaluation, the patient felt that the appliance was stuck in his upper chest and was associated with significant chest discomfort. Examination revealed audible upper airway wheezing, but normal bowel sounds and no abdominal tenderness. Chest X-ray showed a radiopaque foreign body (denture) near the gastroesophageal junction, and emergent endoscopy (EGD) was done, but the appliance had passed beyond the reach of the upper endoscope and was not visualized. Serial abdominal X-rays were performed to observe denture passage through the gastrointestinal (GI) tract where it eventually came to rest in the area of the cecum/ascending colon (Figure 1).

**Figure 2:**
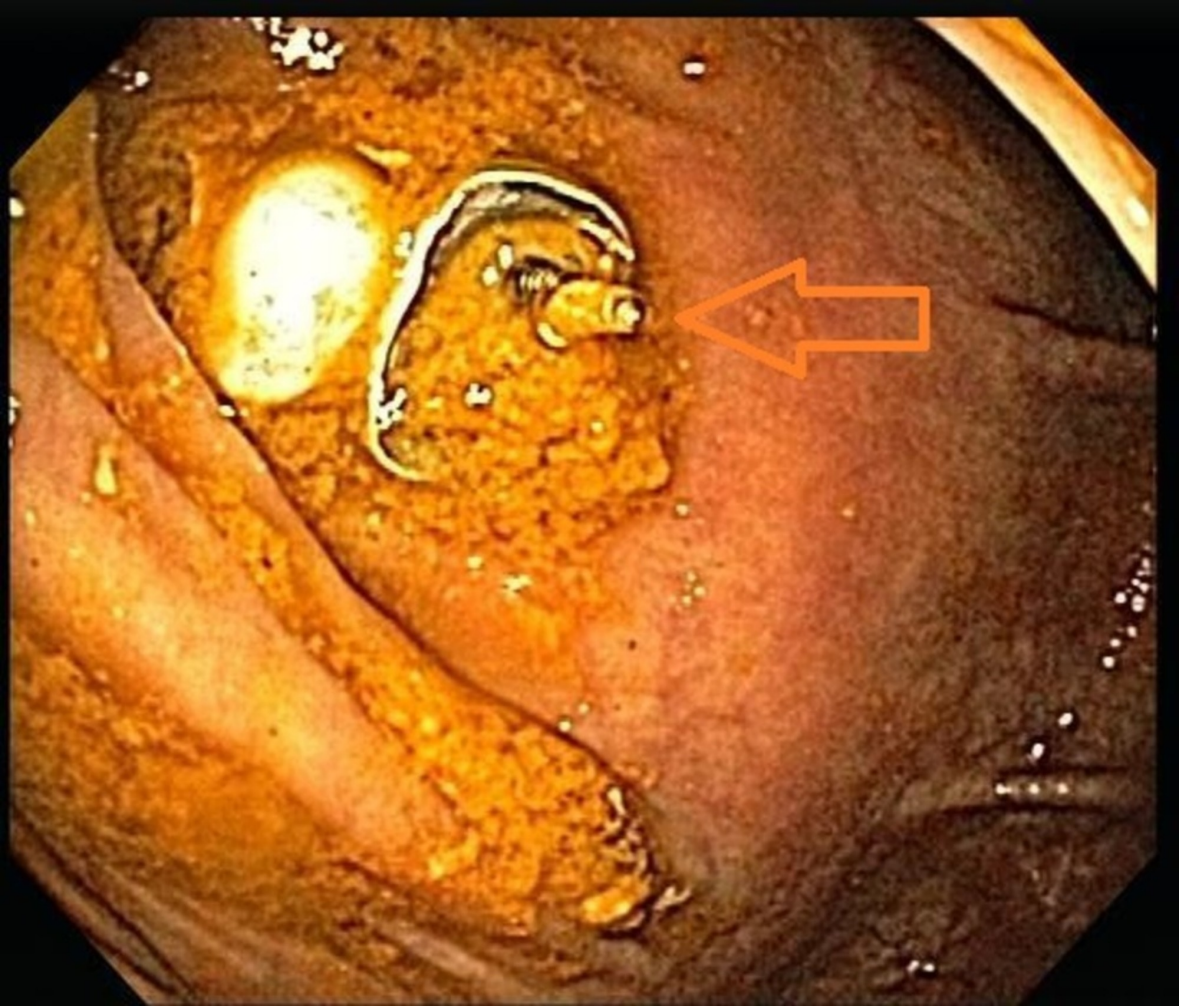
Dental bridge in the ascending colon

There was no further advancement after three days and bowel preparation, and the patient continued to complain of abdominal pain. Therefore, a colonoscopy was performed for removal. On entry into the proximal ascending colon, the appliance was visualized clearly (Figure 2). 

A snare was used to grasp the exposed screw, and it was slowly removed with one attempt. The patient’s abdominal pain resolved post-procedure and was discharged in a stable condition.

## Discussion

The ingestion of foreign bodies is a severe event, common in children and adults with neuropsychiatric disorders that can have significant complications, but it is uncommon to see in healthy patients.

Foreign bodies in the esophagus can produce various symptoms, including dysphagia, drooling, and occasionally airway obstruction. GI foreign bodies produce fewer specific symptoms, including abdominal pain, melena, and hematochezia [4].

Imaging is only performed in patients without signs or symptoms suggestive of esophageal obstruction. In patients without a suspected esophageal obstruction and a history of ingestion of a radiopaque blunt foreign body or if the type of object is unknown, plain radiographs is needed.

More than 80% of ingested foreign bodies pass without the need for intervention. Impaction may occur behind a physiologic/pathologic luminal narrowing. The approach to management depends upon the object ingested, the location, and the patient's clinical status. All foreign bodies in the esophagus require removal within 24 hours. Most foreign bodies that enter the stomach will pass in four to six days, and conservative management is appropriate for most blunt objects in asymptomatic patients. Endoscopic intervention is required for sharp-pointed objects, magnets, blunt objects >5 cm length or >2 cm in diameter, and disk batteries remaining in the stomach longer than 24 hours.

If a foreign body cannot be retrieved endoscopically, daily radiographs should be performed to assess the object’s passage through the GI tract. Surgery is reserved for patients who develop complications (e.g., obstruction, perforation) and for non-progression of a foreign body (a blunt object that remains in the same location distal to the duodenum for more than one week or a sharp foreign body that does not advance radiographically for three consecutive days).

## Conclusions

Ingestion of foreign body is a common clinical problem in children but rare to see in a healthy adult. Foreign body ingestion with its insidious nature may have a complicated course, which may cause serious complications when not correctly managed. Though most of them can be managed conservatively, close monitoring is required to avoid complications associated with foreign body ingestion.
